# Parallel anagenetic patterns in endemic *Artemisia* species from three Macaronesian archipelagos

**DOI:** 10.1093/aobpla/plad057

**Published:** 2023-08-14

**Authors:** Daniel Vitales, Carmen Guerrero, Teresa Garnatje, Maria M Romeiras, Arnoldo Santos, Francisco Fernandes, Joan Vallès

**Affiliations:** Institut Botànic de Barcelona (IBB), CSIC-Ajuntament de Barcelona, Passeig del Migdia s/n, 08038 Barcelona, Catalonia, Spain; Laboratori de Botànica (UB), Unitat Associada al CSIC, Facultat de Farmàcia i Ciències de l’Alimentació-Institut de Recerca de la Biodiversitat (IRBio), Universitat de Barcelona, 08028 Barcelona, Catalonia, Spain; Institut Botànic de Barcelona (IBB), CSIC-Ajuntament de Barcelona, Passeig del Migdia s/n, 08038 Barcelona, Catalonia, Spain; Institut Botànic de Barcelona (IBB), CSIC-Ajuntament de Barcelona, Passeig del Migdia s/n, 08038 Barcelona, Catalonia, Spain; LEAF—Linking Landscape, Environment, Agriculture and Food Research Center & Associated Laboratory TERRA, Instituto Superior de Agronomia (ISA), Universidade de Lisboa, 1340-017 Lisboa, Portugal; Calle Guaidil 16, 38280 Tegueste, Tenerife, Islas Canarias, Spain; Jardim Botânico da Madeira Eng. Rui Vieira, Caminho do Meio Bom Sucesso, Madeira, Portugal; Laboratori de Botànica (UB), Unitat Associada al CSIC, Facultat de Farmàcia i Ciències de l’Alimentació-Institut de Recerca de la Biodiversitat (IRBio), Universitat de Barcelona, 08028 Barcelona, Catalonia, Spain

**Keywords:** Canary Islands, Cape Verde, genetic diversity, Madeira, phylogeography, plant speciation

## Abstract

Anagenetic speciation is an important mode of evolution in oceanic islands, yet relatively understudied compared to adaptive radiation. In the Macaronesian region, three closely related species of *Artemisia* (i.e. *A. argentea*, *A. thuscula* and *A. gorgonum*) are each endemic from a single archipelago (i.e. Madeira, Canary Islands and Cape Verde, respectively), representing a perfect opportunity to study three similar but independent anagenetic speciation processes. By analysing plastid and nuclear DNA sequences, as well as nuclear DNA amount data, generated from a comprehensive sampling in all the islands and archipelagos where these species are currently distributed, we intend to find common evolutionary patterns that help us explain the limited taxonomic diversification experienced by endemic Macaronesian *Artemisia*. Our time-calibrated phylogenetic reconstruction suggested that divergence among the three lineages occurred in a coincidental short period of time during the Pleistocene. Haplotype and genetic differentiation analyses showed similar diversity values among *A. argentea*, *A. thuscula* and *A. gorgonum*. Clear phylogeographic patterns—showing comparable genetic structuring among groups of islands—were also found within the three archipelagos. Even from the cytogenetic point of view, the three species presented similarly lower genome size values compared to the mainland closely related species *A. arborescens*. We hypothesize that the limited speciation experienced by the endemic *Artemisia* in Madeira, Canary Islands and Cape Verde archipelagos could be related to their recent parallel evolutionary histories as independent lineages, combined with certain shared characteristics of seed dispersal, pollen transport and type of habitat.

## Teaser

Anagenetic speciation in oceanic islands—where initial colonizers arrive on an island or archipelago and then diverge into a single new species, but without subsequent taxonomic differentiation—is relatively understudied compared to adaptive radiation. In the Macaronesian region, three closely related species of *Artemisia* (i.e. *A. argentea , A. thuscula* and *A. gorgonum* ) are each endemic from a single archipelago (i.e. Madeira, Canary Islands and Cape Verde, respectively). By analysing plastid and nuclear DNA sequences, as well as nuclear DNA amount data, we found that the three single-endemic species showed similar genetic diversity, phylogeographic structure and cytogenetic patterns. We hypothesize that the limited speciation experienced by the endemic Macaronesian *Artemisia* could be related to their recent parallel evolutionary histories as independent lineages, combined with certain shared characteristics of seed dispersal, pollen transport and type of habitat.

## Introduction

Oceanic islands have been, and still remain, a subject of continuous biological study as they are an inexhaustible source of evolutionary processes (Whittaker *et al*. 2017). Volcanic island archipelagos have been of particular interest, as they harbour a disproportionately high level of biodiversity, much of which has evolved *in situ* from a small number of original transoceanic colonizers (Losos and Ricklefs 2010). Species-rich adaptive radiations arising from animal and plant colonizers are common on oceanic archipelagos around the globe (e.g. Jorgensen and Olesen 2001; Petren *et al*. 2005; Givnish *et al*. 2009). These accelerated speciation events may be driven by geographical isolation, complex geological history and/or a wide variety of ecological niches (Whittaker and Fernández-Palacios 2007). However, some lineages fail to diversify at the same fast level, despite being exposed to the same volcanic stresses as organisms that undergo rapid evolutionary diversification. Anagenetic speciation—where initial colonizers arrive on an island, slowly accumulate genetic variation in a relatively uniform environment and then diverge into a new species, but without subsequent taxonomic differentiation—is an important mode of evolution in oceanic islands (Stuessy *et al*. 2006), yet relatively understudied compared to adaptive radiation.

The Macaronesian region is one of the most exceptional areas to study plant evolution and speciation in oceanic islands. Macaronesia consists of five volcanic archipelagos situated in the North Atlantic Ocean (i.e. Azores, Madeira, Savage Islands, Canary Islands and Cape Verde) and shows wide latitudinal, altitudinal, and climate variation among the 40 islands grouped within these archipelagos, which are in part responsible for the diversity of habitats they host (Fernández-Palacios *et al*. 2011). The flora of the Macaronesian islands is very rich and diverse, with approximately 3337 native species of vascular plants, 25% of which are considered endemic flora of the region (Florencio *et al.* 2021). The Canary Islands, with ca. 573 endemic species of higher plants,are the archipelago that has more plant biodiversity, followed by Madeira with 124, Cape Verde with 68, Azores with 65 and the Savage Islands with 11 endemic species (excluding infraspecific taxa) (Florencio *et al.* 2021 and references therein). Regarding the models of speciation, cladogenesis (i.e. two or more species descended from the same introduction, within an archipelago) has been inferred as the most frequent process in Macaronesian archipelagos. Nevertheless, it has been estimated that anagenetic diversification originated from 16% (Canary Islands) to 48% (Madeira archipelago) of endemic flora in these islands (Stuessy *et al*. 2006).

Three species of *Artemisia* endemic to the Macaronesian archipelagos have been described to date: *A. argentea* L’Hér., endemic to Madeira, *A. thuscula* Cav. (synonym: *A. canariensis* (Besser) Less.), endemic to the Canary Islands, and *A. gorgonum* Webb, endemic to Cape Verde. These Macaronesian taxa have been considered the vicariant species of *A. arborescens* L., a species occurring in the Mediterranean region, but not present in these oceanic islands, where the habitats it usually colonizes—rocky scrubland influenced by humid sea winds—are occupied by the above-cited local endemics (Garcia *et al*. 2006). There are no other known endemic *Artemisia* species in these three groups of islands nor the remaining Macaronesian archipelagos, the Azores and the Savage Islands. *Artemisia ramosa* C.Sm. in Buch, a species of subgenus *Seriphidium* and part of the taxonomically intricate *A. herba-alba* Asso group, was considered endemic to the Canary Islands, but has also been reported from Morocco (Charpin and Vallès 1985). Also in the Canary Islands, *A. reptans* C.Sm. in Buch (subgenus *Artemisia*) is a native but non-endemic species, also present in Northwest Africa (Gobierno de Canarias 2023). The reports of this latter species from south-east Spain correspond to the Iberian endemic *A. aethiopica* L. (*A. lucentica* O.Bolòs, Vallès & Vigo; *A. hispanica* Lam.; Benedí 2019), which was erroneously merged with *A. reptans* in Flora Europaea (Tutin *et al.* 1976). In Madeira, *A. verlotiorum* Lamotte (subgenus *Artemisia*) is considered an introduced taxon (Borges *et al.* 2008). In the Azores, there has been reported the presence of *A. absinthium* L. – a phylogenetically close species to the Macaronesian *Artemisia* group—as well as *A. arborescens* and *A. dracunculus* L., but only as introduced taxa (Portal da Biodiversidade dos Açores 2023).

The three endemic Macaronesian *Artemisia* species, as well as *A. arborescens*, show resembling biological characteristics. According to the information available in the floras of the regions where these species are distributed (Tutin *et al.* 1976; Bolòs and Vigo 1990; Press 1994; Brochmann *et al.* 1997; Bramwell and Bramwell 2001; Pignatti 2018), most of the ecological and morphological characters are coincident or overlapping among the taxa **[see Supporting Information—Table S1]**. From the reproductive point of view, important shared traits in these species, such as wind pollination and large production of small achenes lacking pappus, are typical of the genus (Vallès *et al.* 2011). Webb (1849) also noted the morphological similarities between *A. gorgonum* and *A. thuscula*, observing differences in involucre bracts and flower traits (i.e. shape of the anthers and stigmas). According to the phylogenetic reconstructions of *Artemisia* performed by Hobbs and Baldwin (2013), Malik *et al*. (2017) and Jiao *et al.* (2023), the three endemic Macaronesian species of *Artemisia* (i.e. *A. argentea, A. thuscula* and *A. gorgonum*) constitute a monophyletic clade within the subgenus *Absinthium*, together with the continental—including continental islands as well—species *A. arborescens*. The evolutionary relationships among species within this clade differ between those former phylogenetic studies. However, they have in common that none of them infers *A. arborescens* as the sister species of the Macaronesian endemics, suggesting multiple colonization events from the continent. Everything considered, this Mediterranean–Macaronesian group of closely related species—showing very similar characteristics in terms of morphology, ecological preferences, reproduction strategies and life cycle—represents a perfect opportunity to study anagenetic speciation processes replicated in the three mentioned archipelagos.

In this paper, we will investigate the evolutionary patterns of the three endemic Macaronesian *Artemisia* by analysing plastid and nuclear DNA sequences, as well as nuclear DNA amount data, generated from a comprehensive sampling in all the islands and archipelagos where these species are currently distributed. More specifically, these data obtained at the population level will be employed to i) delineate from a molecular point of view the taxonomic identity—based to date just on morphological characters and geographic distribution—of the three species endemic from Madeira, Canary Islands and Cape Verde. Our genetic data will also be analysed to ii) infer the evolutionary relationship among the endemic Macaronesian *Artemisia,* examining the colonization history of these archipelagos. From a phylogeographic point of view, in all cases of anagenesis evaluated in recent studies, no geographical patterning of the observed genetic variation has been reported among populations of the endemic island species (Stuessy *et al*. 2014; Takayama *et al*. 2015). In this context, we will iii) explore whether this general lack of phylogeographic structure in insular species originated anagenetically is also fulfilled by the Macaronesian endemic *Artemisia* or whether genetic diversity of these species shows phylogeographic patterns comparable to others previously inferred from the same archipelagos. Finally, considering—the genetic results obtained—together with available knowledge on the biological attributes of the species—we will try to iv) explain the limited taxonomic diversification experienced by this group of insular endemic plants.

## Material and methods

### Sampling and sequencing procedures

**Figure 1.**
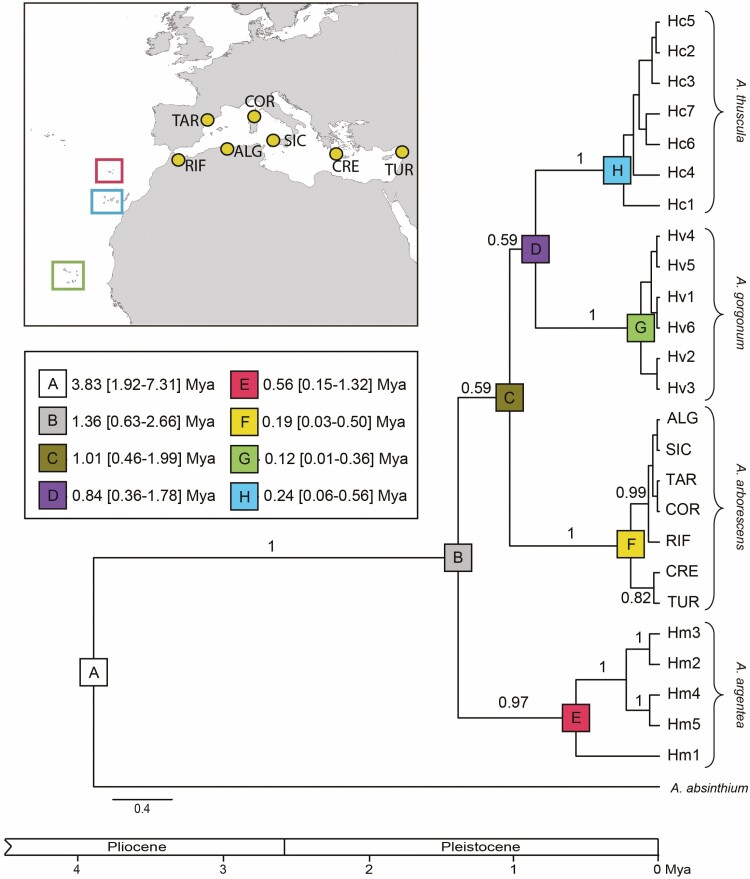
Time-calibrated phylogenetic reconstruction of endemic Macaronesian *Artemisia* species and *A. arborescens* based on Bayesian inference of the combined plastid regions. Posterior probability (PP) values are shown above branches. The map indicates the geographic distribution of each Macaronesian species as well as the location of *A. arborescens* samples included in the study.

We collected from the field plant material of 33 populations belonging to the three Macaronesian endemic species of *Artemisia***[see Supporting Information—Table S2]**. Sequencing of plastid DNA (ndhC-trnV, rpl32-trnL and rps16-trnK) was performed for 141 individuals from 29 populations (5 individuals per population, excepting for a locality in Madeira where only one individual was obtained) of *A. thuscula*, *A. argentea* and *A. gorgonum* (Table 1). Sequencing of nuclear ribosomal DNA (ITS and ETS) was performed for 22 populations from all species and islands (one individual per population) as a screening of variability in these multiple-copy nuclear markers. Samples from all the islands within each archipelago where these species are distributed were included. Vouchers of each population studied were prepared and deposited in the herbarium BCN, of the Centre de Documentació de Biodiversitat Vegetal, Universitat de Barcelona. Seven accessions of *A. arborescens*—most of them collected by our team—from localities around the distribution range of the species in the Mediterranean Basin (Fig. 1) were also sampled from BC (Institut Botànic de Barcelona, CSIC-Ajuntament de Barcelona) and BCN (Universitat de Barcelona) herbaria. Collection details, voucher information and codes of plant material are listed in **Supporting Information—Table S2**.

**Table 1.** Sampling information, codes and haplotypes of endemic Macaronesian *Artemisia* populations studied from a genetic point of view.

Taxon	Code	Collection site	*N*	Haplotype
*Artemisia thuscula* Cav.	C-1*	Canary Islands, Tenerife. Icod de los Vinos	5	Hc2(5)
	C-2*	Canary Islands, Tenerife. Punta de Teno	5	Hc2(5)
	C-3*	Canary Islands, Tenerife. Anaga	5	Hc2(5)
	C-4*	Canary Islands, Tenerife. Güimar	5	Hc2(5)
	C-5*	Canary Islands, La Gomera. Tunnel of La Culata	5	Hc3(5)
	C-6*	Canary Islands, La Gomera. Benchijigua	5	Hc3(5)
	C-7*	Canary Islands, El Hierro. La Peña	5	Hc3(2), Hc4(2), Hc6(1)
	C-8	Canary Islands, El Hierro. Los Llanillos	5	Hc3(4), Hc6(1)
	C-9	Canary Islands, El Hierro. El Pinar	5	Hc3(5)
	C-10	Canary Islands, La Palma. Puntallana	5	Hc3(5)
	C-11*	Canary Islands, La Palma. Mazo	5	Hc5(5)
	C-12*	Canary Islands, La Palma. Fuencaliente	5	Hc3(4), Hc7(1)
	C-13*	Canary Islands, Gran Canaria. Andén Verde	5	Hc2(5)
	C-14*	Canary Islands, Gran Canaria. Ravine of Guayadeque	5	Hc1(5)
	C-15	Canary Islands, Gran Canaria. Los Tiles de Moya	5	Hc1(1), Hc2(4)
	C-16	Canary Islands, Tenerife. Tejina mountain	5	Hc2(5)
*Artemisia argentea* L’Hér.	M-1*	Madeira, Desertas Islands. Plain top of Ilhéu Chão	5	Hm1(5)
	M-2*	Madeira, Madeira. Quinta Grande	5	Hm4(5)
	M-3*	Madeira, Madeira. Ponta do Pargo	5	Hm2(5)
	M-4	Madeira, Porto Santo. Pico Branco	5	Hm1(5)
	M-5*	Madeira, Porto Santo. Os Morenos	5	Hm1(5)
	M-6*	Madeira, Porto Santo. Ilhéu do Farol	5	Hm1(4), Hm3(1)
	M-7*	Madeira, Madeira. Pico Cidrão, down to Pico do Gato	1	Hm5(1)
*Artemisia gorgonum* Webb	V-1*	Cape Verde, Santo Antão. Natural park of Moroços	5	Hv1(5)
	V-2*	Cape Verde, Santo Antão. Natural park do Topo de Corroa	5	Hv2(4), Hv3(1)
	V-3	Cape Verde, Santo Antão. Pico da Cruz	5	Hv3(5)
	V-4*	Cape Verde, Santiago. Praia, material acquired in a local market, from the Pico da Antónia	5	Hv4(5)
	V-5*	Cape Verde, Fogo. Floresta do Monte Velha	5	Hv4(3), Hv5(1), Hv6(1)
	V-6*	Cape Verde, Fogo. Topo da Bordera	5	Hv4(5)

* Populations included in the screening analyses of nuclear ribosomal DNA variability.

Total genomic DNA was extracted following the CTAB method of Doyle and Doyle (1987), as modified by Soltis *et al*. (1991), from silica gel-dried leaves collected in the field and herbarium material. Plastid intergenic DNA regions and nuclear ribosomal DNA regions were amplified and sequenced for *A. thuscula*, *A. argentea* and *A. gorgonum* samples, as well as for herbarium accessions of *A. arborescens*, according to the procedures described in Malik *et al*. (2017). One sample of *A. absinthium* collected in the Pyrenees and deposited in the herbarium BCN was also sequenced as outgroup for phylogenetic analysis. The amplification procedure was performed using the following primers specified in Shaw *et al*. (2007): ndhC and trnV(UAC)x2 primers for ndhC-trnV, rps16 × 2F2 and trnk(UUU)x1 for rps16-trnK and rpl32-F and trnL(UAG) for rpl32-trnL. Amplification primers of nuclear ribosomal DNA (nrDNA) markers were ITS1 and ITS4 for ITS (White *et al*. 1990) and ETS1f and 18S2L for ETS (Baldwin and Markos 1998; Linder *et al*. 2000). Polymerase chain reaction (PCR) was performed by using either Flexcycler (Analytikjena) or S1000TM Thermal Cycler (Bio-Rad thermal) cyclers in a 25 μL volume. Direct sequencing of the amplified DNA segment was performed with the Big Dye Terminator Cycle Sequencing v3.1 (PE Biosystems, Foster City, CA, USA). Nucleotide sequencing was carried out at the Unitat de Genòmica, Centres Científics i Tecnològics, Universitat de Barcelona (CCiTUB) on an ABI PRISM 3700 DNA analyzer (PE Biosystems). The DNA sequences were visualized with Chromas Lite 2.01 (Technelysium PTy, Tewantin, Queensland, Australia) and subsequently assembled with Geneious Prime (Biomatters Ltd.), aligned with MAFFT v7 (Katoh *et al*. 2002), and corrected manually. GenBank accession numbers are provided in **Supporting Information—Table S3**.

### DNA sequencing analyses

Plastid haplotypes were determined from nucleotide substitutions in a combined data set that included the plastid regions ndhC-trnV, rpl32-trnL and rps16-trnK. To increase the information contributed by plastid DNA, indels were codified with FastGap v.1.2 (Borchsenius 2009) and treated as single-mutation events. The genetic variability existing in Macaronesian endemic *Artemisia* was calculated using the program DnaSP 6 (Rozas *et al*. 2017). The number of polymorphic sites, nucleotide diversity (p), number of haplotypes (h) and haplotype diversity (Hd) were calculated for groups of samples according to geographical origin (i.e. islands and archipelagos). The evolutionary relationships among haplotypes from each Macaronesian species were inferred based on parsimony TCS networks constructed using PopArt (Leigh and Bryant 2015) with default settings. Haplotype spatial genetic structure was further analysed with SAMOVA2 (Dupanloup *et al.* 2002), carrying out a simulated annealing approach to identify populations clusters in each Macaronesian species. We explored *K* values from 2 to 7 (excepting for *A. argentea* and *A. gorgonum*, where the maximum number of groups was 6 and 5, respectively), starting from 10 000 random initial conditions for each simulation, and chose the number of groups that gave the highest Δ*F*_CT_. Finally, we conducted analysis of molecular variance (AMOVA) in Arlequin v3.5 with 10 000 replicates (Excoffier and Lischer 2010) to measure variation among populations and to test the genetic differentiation between groups of populations according to: i) SAMOVA clustering and ii) main geographic units (i.e. islands within the archipelagos).

A matrix was constructed with a sequence of each of the Macaronesian haplotypes, as well as the sequences from seven *A. arborecens* accessions and *A. absinthium* as outgroup, to infer their phylogenetic relationship. Evolutionary models were selected with jModeltest 2.1.10 (Darriba *et al*. 2012) under the Akaike Information Criterion, AIC (Akaike 1974). The chosen model (GTR + G) was subsequently used to perform Bayesian MCMC analyses with BEAST 1.8.4 (Drummond *et al*. 2012). We also calculated the divergence time of all haplotypes using a strict-clock model. No fossil records or unambiguous biogeographic events isolating distinct populations of Macaronesian endemic *Artemisia* are available to calibrate the plastid DNA substitution rate. Therefore, we estimated divergences within haplotypes using the average rate for non-coding regions of cpDNA [1.52 × 10^9^ s/s/y (1.0 × 10^9^ s/s/y as the lower limit and 3.0 × 10^9^ s/s/y as the upper limit); Wolfe *et al*. 1987; Richardson *et al*. 2001]. Indels were treated as missing data in time-calibrated phylogenetic analyses. Four Markov chains Monte Carlo (MCMC) were run for 5 · 10^7^ generations, sampling every 1000 generations. The program TRACER version 1.6 (Rambaut *et al*. 2014) was used to check the convergence and mixing of each parameter. Posterior probabilities (PP) were estimated through the construction of a maximum clade credibility (MCC) tree. The output trees were visualized and edited with FigTree v. 1.2.2. (http://tree.bio.ed.ac.uk/software/figtree).

Nuclear ribosomal DNA sequences were first edited to code intra-individual nucleotide polymorphisms—visualized in the chromatograms as two peaks close to equal in height—according to IUPAC terminology (W, R, Y, S, K and M). Sequences were then phased using the Bayesian statistical method implemented in PHASE 2.1.1 (Stephens and Donnelly 2003) with the parameters by default. To visualize the relationships among the phased sequences, POPART was used for reconstructing a parsimony TCS network of the ribotypes. However, PHASE might estimate erroneous alleles when input data are not diploid (ITS and ETS occur as multicopy tandem repeats), so network structure using ‘phased’ alleles might be inaccurate. Therefore, we also constructed a neighbour‐net network in Splitstree v4.14.8 (Huson and Bryant 2006) using direct sequences with intra-individual polymorphisms scored according to IUPAC terminology.

### Nuclear DNA content analyses

The nuclear DNA amount or genome size of Macaronesian endemic *Artemisia* was estimated using flow cytometry at the Unitat de Citometria, Centres Científics i Tecnològics, Universitat de Barcelona (CCiTUB), following the protocol described in Garcia *et al*. (2006). Five individuals per population and two replicates per individual were studied in four populations of *A. argentea*, five populations of *A. thuscula* and three populations of *A. gorgonum***[see Supporting Information—Table S2]**. As for the sequencing procedures, samples from all the islands where these species are distributed were included in genome size analyses. Vouchers of each population were also deposited in the herbarium BCN. We compared the measures of qDNA among the Macaronesian species to check whether significant differences occurred between taxa from different archipelagos. These genome size data were also compared with qDNA values of *A. arborescens* sampled from mainland and continental islands published in Garcia *et al*. (2006). Statistical analyses were performed using the software R v.3.5.2 (R Core Team 2018). First, we analysed the homogeneity of variance (Bartlett test) and performed the Shapiro–Wilk test to check the distribution of residues, both for the whole dataset and for the Macaronesian dataset. A nonparametric Wilcoxon test was used to test the possible statistically significant difference between the amount of DNA in populations of oceanic islands and the mainland species *A*. *arborescens*. To analyse possible differences in the amount of DNA between the species of the three archipelagos, a one-way ANOVA test with a post hoc Tukey’s test for comparison of means between archipelagos was carried out.

## Results

### Genetic data

The sequences of ndhC-trnV, rpl32-trnL, rps16-trnK intergenic spacers obtained from *A. argentea, A. thuscula* and *A. gorgonum* samples were aligned in three matrices (one per region) containing 841, 905 and 725 nucleotides, respectively. All these plastid DNA regions showed a moderate level of polymorphism among the 141 specimens of Macaronesian endemic *Artemisia* analysed in this study. Specifically, 13, 16 and 6 polymorphic segregating sites (including indels) were, respectively, observed in ndhC-trnV, rpl32-trnL, rps16-trnK regions. Based on analysis of the concatenated matrix—including nucleotide polymorphisms and indels—we were able to identify 18 haplotypes across the 29 Macaronesian populations (Table 1). According to the Bayesian phylogenetic inference summarized in the MCC tree (Fig. 1), these haplotype sequences were grouped in three supported lineages corresponding to the three Macaronesian endemic *Artemisia* species. The seven accessions of *A. arborescens* included in the phylogenetic analysis were also clustered in a fully supported lineage. Internal nodes of the MCC tree did not show statistical support, but the reconstructed topology grouped *A. thuscula* and *A. gorgonum* in the same branch. The MCC tree topology showed that *A. arborescens* could be the sister lineage of these Canarian and Cape Verde species, but the splitting node presents no statistical support (PP = 0.59). The species from Madeira, *A. argentea*, is represented in an early diverging branch of the MCC tree, this position also lacking statistical support. According to the strict-clock time calibration of the tree (Fig. 1), the Macaronesian–Mediterranean lineage (i.e. the clade including *A. arborescens* and the three Macaronesian endemic species) diverged from its sister *A. absinthium* lineage ca. 3.83 million years ago (Mya). Diversification among species/archipelagos lineages was estimated to occur 1.36 Mya (95% of Highest Posterior Density, HPD, 0.63–2.66 Mya) to 0.84 Mya (95% HPD 0.36–1.78 Mya) ago. Genetic differentiation within the species started from ca. 0.56 Mya ago in *A. argentea* to ca. 0.12 Mya ago in *A. gorgonum*.

The results of haplotype and nucleotide variability analyses revealed similar genetic diversity levels among Macaronesian taxa (Table 2). *Artemisia thuscula* is the species showing the largest number of haplotypes (*H* = 7), while *A. gorgonum* from Cape Verde shows the highest haplotype diversity (Hp = 0.749). In contrast, *A. argentea* sequences present the largest number of segregating sites (*S* = 9) and the highest nucleotide diversity (π = 0.00136), despite showing the lowest number of haplotypes (*H* = 5) as well as the lowest haplotype diversity (Hp = 0.589). Most populations harbour a single haplotype, with only one out of seven populations in *A. argentea*, four out of 16 populations in *A. thuscula* and two out of six populations in *A. gorgonum* presenting some degree of haplotype diversity (i.e. two or three haplotypes within the same population).

**Table 2.** Genetic variability values based on plastid DNA sequences for the three Macaronesia endemic *Artemisia* species analysed in the study and the islands where they are distributed.

	#P	*N*	Hp	Hd	S	π
***A. argentea***	**7**	**31**	**5**	**0.589**	**9**	**0.00136**
Madeira	3	11	3	0.636	5	0.001
Porto Santo	3	15	2	0.133	4	0.00023
Desertas Islands	1	5	1	0	0	0
***A. thuscula***	**16**	**80**	**7**	**0.676**	**7**	**0.00044**
Gran Canaria	3	15	2	0.514	2	0.00044
Tenerife	5	25	1	0	0	0
La Gomera	2	10	1	0	0	0
La Palma	3	15	3	0.562	2	0.00026
El Hierro	3	15	3	0.457	2	0.00021
***A. gorgonum***	**6**	**30**	**6**	**0.749**	**5**	**0.00049**
Santo Antão	3	15	3	0.705	2	0.00037
Fogo	2	10	3	0.378	2	0.00017
Santiago	1	5	1	0	0	0

#P, number of sampling sites; *N*, number of individuals; Hp, number of haplotypes; Hd, haplotype diversity; *S*, number of segregating sites; π, nucleotide diversity.

**Figure 2.**
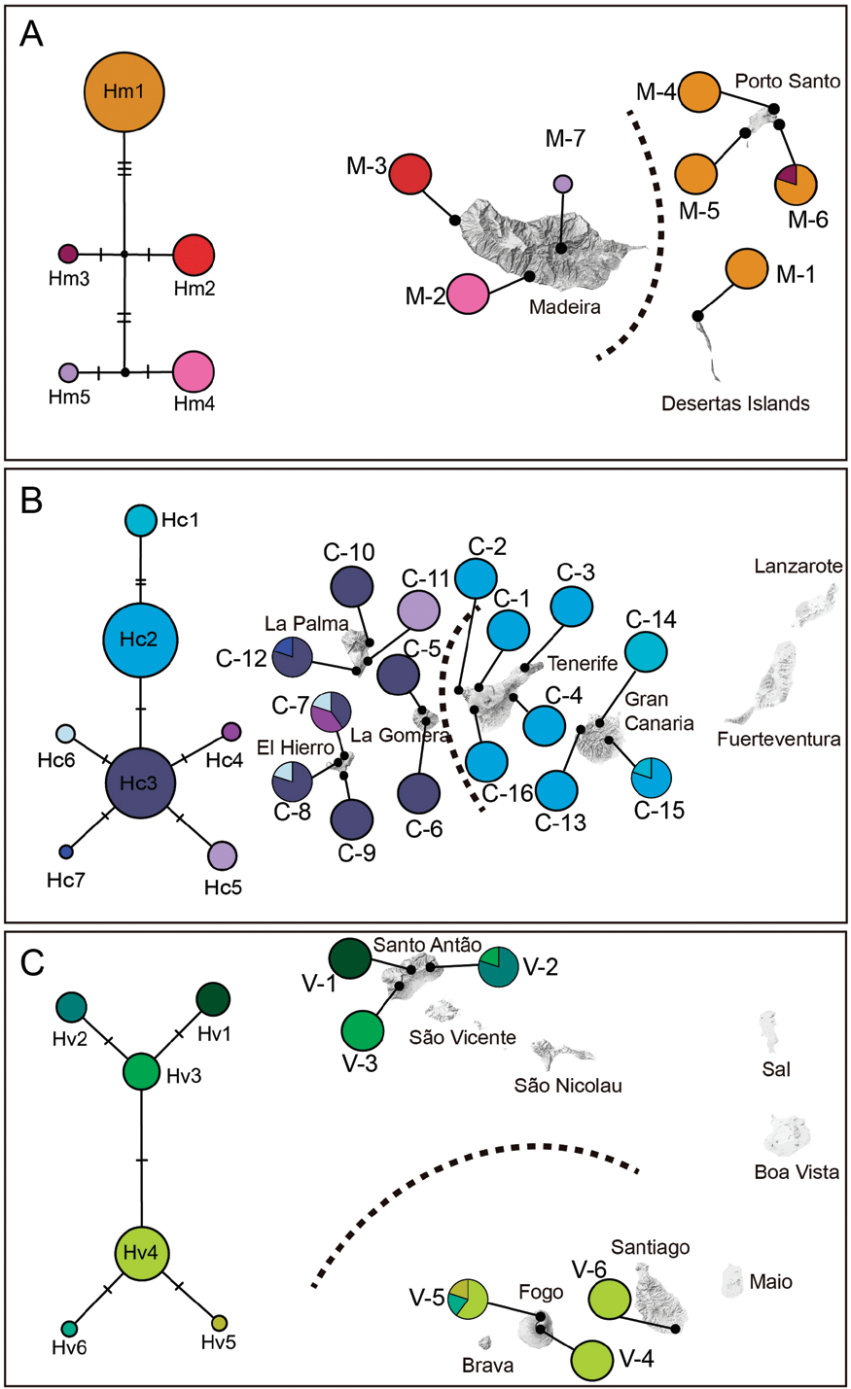
Statistical parsimony network and geographic distribution of haplotypes found in each endemic Macaronesian *Artemisia* species. (A) *Artemisia argentea*—Madeira; (B) *Artemisia thuscula*—Canary Islands; (C) *Artemisia gorgonum—*Cape Verde. The size of the circles in the networks represents the number of individuals and crossing bars indicate one base mutation distance and black dots represent unsampled inferred haplotypes. Discontinuous lines in the maps indicate genetic boundaries as inferred by SAMOVA analyses.

The parsimony haplotype networks, together with the geographic distribution of haplotypes, showed clear phylogeographic patterns in each Macaronesian species (Fig. 2). Haplotypes in the networks were generally connected by one or two mutation steps, excepting in *A. argentea*, where haplotypes were separated by three to five mutation steps. In all species, frequent haplotypes (e.g. Hm1 in 19 individuals of *A. argentea*; Hc2 or Hc3 in, respectively, 34 and 30 individuals of *A. thuscula*; Hv4 in 18 individuals of *A. gorgonum*) were combined with relatively rare haplotypes (found in one to five individuals per species). Phylogeographic pattern in *A. argentea* showed haplotype Hm1 widely distributed in Porto Santo and Desertas Islands, while populations in Madeira Island exclusively presented other three haplotypes (Hm2, Hm4 and Hm5). In *A. thuscula*, haplotype Hc2 dominated in populations from Tenerife and Gran Canaria (34 out of 40 individuals), while Hc3 was the main haplotype in La Gomera, La Palma and El Hierro (30 out of 40 individuals). The rest of Canarian haplotypes were less frequent, mainly found in El Hierro or La Palma and being always exclusive from a single island. In *A. gorgonum*, northern Santo Antão Island harboured three exclusive haplotypes (Hv1, Hv2 and Hv3), each of them dominating in a different population. In contrast, in southern islands of Santiago and Fogo, one main haplotype (Hv4) was widely distributed in all three studied populations, the other two haplotypes Hv5 and Hv6 being only found in a couple of individuals from Fogo.

Spatial genetic analyses of plastid DNA haplotypes using SAMOVA indicated that the largest increase of *F*_CT_ values occurred at *K* = 2 for the three Macaronesian species **[see Supporting Information—Figure S1]**. In all cases, the genetic structure showed by *K* = 2 clustered populations according to a geographic pattern. For larger values of *K*, the same clustering pattern recovered for K2 was always maintained, but each time one additional population split into an independent group. Therefore, we considered *K* = 2 as the most informative number of groups to explain phylogeographic structure of endemic Macaronesian *Artemisia*. In *A. argentea*, the genetic structure showed by *K* = 2 clustered the populations from Porto Santo and Desertas in one group and the populations from Madeira in another group **[see Supporting Information—Figure S2A]**. SAMOVA analysis for *A. thuscula* split populations from Gran Canaria and Tenerife in one genetic group and populations from La Gomera, La Palma and El Hierro in a separate genetic group **[see Supporting Information—Figure S2B]**. Regarding *A. gorgonum*, the spatial genetic analyses differentiated one group for Santo Antão populations and another group for Fogo and Santiago populations **[see Supporting Information—Figure S2C]**. The results of AMOVA analyses studying the partitioning of genetic diversity are summarized in **Supporting Information—Table S4**. Genetic differences among *K* = 2 clusters defined by SAMOVA explained a significant proportion of the variance in all species (75.22 % in *A. argentea*; 65.54 % in *A. thuscula*; 57.58 % in *A. gorgonum*), while genetic differences among islands represented lower percentage values in all cases.

Regarding nuclear DNA regions, we were only able to obtain good quality sequences from short fragments of ITS (202 bp) and ETS (346 bp) nuclear DNA regions. From that nucleotide positions onwards, double messy signals in the sequence chromatograms made impossible the base calling. Multiple intra-individual nucleotide polymorphisms visualized in the chromatograms as two peaks close to equal in height were detected along the fragments of the sequences that could be correctly read. After aligning these nrDNA sequences, 11 intra-individual polymorphic sites for ITS and 10 intra-individual polymorphic sites for ETS were counted. Apart from these polymorphic sites showing sequence variation within individuals, no other genetic variability between the samples was detected. Parsimony TCS network **[see Supporting Information—Figure S3]** showed a complex structure where ribotypes from the different species appeared intermingled without a clear taxonomic pattern. Neighbour-net network **[see Supporting Information—Figure S4]** showed better-defined groups corresponding to the taxonomic assignation of the samples, but sequences from different species were also intermixed in largely reticulated groups.

### Nuclear DNA content

Table 3 displays the nuclear DNA contents of the Macaronesian species that have been studied in this work. The 2C values of *A. argentea, A. thuscula* and *A. gorgonum* are basically the first obtained in natural populations, as the data had so far come from specimens grown in botanical gardens—with only one accession per species—except for one population of *A. thuscula* from Tenerife (Torrell and Vallès 2001; Garcia *et al*. 2006). The genome size values obtained in this study are similar, although slightly lower, to the ones reported in the mentioned works (Torrell and Vallès 2001; Garcia *et al*. 2006). **Supporting Information—Table S5** displays the genome size information of the Macaronesian species together with data from continental *A. arborescens* reported by Garcia *et al*. (2006). Macaronesian species present average values of DNA amount (10.22 pg) lower than the continental *A. arborescens* (11.11 pg). These differences in genome size between continental and Macaronesian species were statistically significant (*P* < 0.05, *W*_12,25_ = 295). There were also small but significant differences in the amounts of DNA among the Macaronesian species (*P* < 0.05, *F*_2,9_ = 8.118). Tukey test revealed that the populations of *A. gorgonum* (Cape Verde) presented an average genome size (10.52 pg) significantly higher than those of *A. thuscula* (Canary Islands; 10.09 pg) and *A. argentea* (Madeira; 10.16 pg). Populations of Canary Islands and Madeira did not show significant differences among them.

**Table 3.** Nuclear DNA content of the *Artemisia* populations studied, one per island where each taxon is present.

Taxon	Code	Island	2C^1^ (pg)	HPCV^1^ (*Artemisia*)	HPCV^1^ (*Pisum*)
*A. argentea*	M-1	Madeira, Desertas Islands, Ilheu Chão	10.25 (0.23)	3.03 (0.62)	3.05 (0.32)
*A. argentea*	M-2	Madeira, Madeira	10.24 (0.06)	2.37 (0.99)	2.82 (0.40)
*A. argentea*	M-4	Madeira, Porto Santo	10.09 (0.21)	3.20 (1.45)	3.11 (0.92)
*A. argentea*	M-6	Madeira, Porto Santo, Ilheu de Cima	10.07 (0.36)	4.14 (5.32)	3.32 (1.34)
*A. thuscula* ^*^	C-17	Canary Islands, Tenerife	10.02 (0.30)	4.15 (0.78)	3.49 (0.64)
*A. thuscula*	C-6	Canary Islands, La Gomera	10.22 (0.21)	3.86 (0.50)	3.36 (0.64)
*A. thuscula*	C-9	Canary Islands, El Hierro	10.24 (0.30)	4.04 (0.42)	3.29 (0.53)
*A. thuscula* ^*^	C-18	Canary Islands, La Palma	10.07 (0.29)	4.26 (1.46)	3.71 (0.38)
*A. thuscula*	C-15	Canary Islands, Gran Canaria	9.91 (0.11)	3.79 (0.45)	3.75 (0.47)
*A. gorgonum*	V-3	Cape Verde, Santo Antão	10.27 (0.05)	3.42 (0.31)	2.72 (0.32
*A. gorgonum* ^*^	V-7	Cape Verde, Santiago	10.60 (0.18)	2.92 (0.50)	2.90 (0.23)
*A. gorgonum* ^*^	V-8	Cape Verde, Fogo	10.69 (0.10)	2.97 (0.46)	2.99 (0.55)

^1^2C and HPCV (half peak coefficient of variation): mean value (SD) of 10 samples from five individuals with two replicates per individual.

^*^These samples were not included in sequencing analyses. Additional details are found in Table S2.

## Discussion

### Colonization of Macaronesia and Speciation of Endemic *Artemisia* Species

Our phylogenetic analyses based on plastid DNA sequences—including a comprehensive sampling in all islands where endemic Macaronesian *Artemisia* are distributed—confirmed that *A. argentea*, *A. thuscula* and *A. gorgonum* constitute well-defined taxonomic units from a genetic point of view, each species being circumscribed to a single archipelago (i.e. Madeira, Canary Islands and Cape Verde, respectively). These endemic *Artemisia* taxa represent an outstanding example of independent anagenetic speciation processes (*sensu*Stuessy *et al.* 2006) that occurred in three archipelagos of Macaronesia within the same genus. However, low resolution at internal nodes of the cladogram prevented a clear reconstruction of their colonization and diversification histories. Our unresolved phylogenetic inference does not allow to explain whether Macaronesian species are the result of a single or multiple colonization events from the mainland or whether *A. arborescens* can be considered the sister species of the Macaronesian clade. The analyses of genome size evidence that Macaronesian species (*A. argentea*, *A. thuscula* and *A. gorgonum*) show a 2C value significantly lower than *A. arborescens* populations, although all those taxa share the chromosome number 2n = 18 (http://ccdb.tau.ac.il/home). In contrast, *A. arborescens* from continental islands show higher 2C values than on the mainland (Garcia *et al*. 2006). These results could suggest that endemic Macaronesian *Artemisia* species constitute a separate evolutionary group from their continental counterpart, *A. arborescens*. However, Suda *et al*. (2003) postulated that selective pressures acting on the archipelagos of Macaronesia have favoured the independent emergence of smaller values of 2C species in islands compared to mainland species. Therefore, as inferred in *Cheirolophus* (Hidalgo *et al*. 2017) or in *Crithmum maritimum* L. (Roxo *et al.* 2023), the observed differences in genome size values among the continental *A. arborescens* and the Macaronesian *Artemisia* species could be better related to selection linked to insular condition (e.g. smaller genomes use fewer resources and show reduced genetic instability) than to the lineage divergence patterns of the group.

As in our results based on plastid DNA data, previous phylogenetic reconstructions of *Artemisia* using nuclear DNA sequences (Malik *et al*. 2017) also showed low resolution on the nodes where lineages of endemic Macaronesian *Artemisia* and *A. arborescens* split. In a recent phylogenomic study of *Artemisia* (Jiao *et al.* 2023), the evolutionary relationship among Macaronesian *Artemisia* and *A. arborescens* was better resolved, but coalescent analyses also revealed poor resolution within this clade. Unresolved topologies at these points of the cladograms suggest that divergence among those lineages could have occurred in a short time span, hindering the gradual accumulation of mutations that enable molecular phylogenetic inferences. Indeed, according to the strict-clock time calibration of our tree (Fig. 1), the splits among the four lineages leading to the Mediterranean *A. arborescens* and the Macaronesian *A. argentea*, *A. thuscula* and *A. gorgonum* were inferred to take place during the Pleistocene in just ca. 0.5 million years (My). Numerous biogeographic studies have proposed that drastic changes in sea level during specific periods of the Pleistocene could have led to the emergence of seamounts facilitating the colonization of Macaronesian archipelagos (e.g. Fernández-Palacios *et al*. 2011; Rijsdijk *et al*. 2014). Certain Macaronesian *Artemisia* populations (e.g. *A. argentea* populations from Ilhéu Chão, in the Desertas Islands of Madeira) occur on very arid and near sea-level habitats, potentially similar to those found in the emerged seamounts. Therefore, we can speculate that the ancestors of Macaronesian *Artemisia* species could have taken advantage of narrow windows of opportunity during emergence of paleo-islands to colonize in parallel the different archipelagos at a similar time.

Genetic variability analyses for the three endemic Macaronesian *Artemisia* showed very similar values among species both in terms of haplotype and nucleotide diversity (Table 3). In anagenetic speciation processes, immigrant populations are expected to accumulate genetic variation over time through mutation and recombination (Stuessy *et al*. 2014). Comparing concentrations of haplotype diversity among areas may be indicative of residence times along the extant distribution range (e.g. Mairal *et al*. 2015; Durán *et al*. 2020; Coello *et al*. 2021). As proposed by Ben-Menni Schuler *et al*. (2021), the absence of a gradient of genetic diversity through Macaronesian archipelagos would disprove that colonization had occurred in a slow stepping stone model (from more diverse to less diverse areas). More likely, in the case of endemic *Artemisia*, similar levels of genetic variability at the three involved archipelagos support the hypothesis that the split between the three Macaronesian lineages could have been completed in a relatively short period of time. Alternatively, but without being exclusive of the previous hypothesis, multiple colonization waves and hybridizations among Macaronesian *Artemisia* lineages (as well as with *A. arborescens* lineage) during their early evolutionary history at Pleistocene could have blurred the phylogenetic signal of molecular markers, homogenizing the genetic diversity levels as well among the species (Caujapé-Castells *et al*. 2017). The large amount of intra-individual nucleotide polymorphisms detected in DNA sequence chromatograms of ITS and ETS markers could be interpreted as evidence of these past hybridization processes (e.g. Nieto Feliner *et al.* 2004; Osuna-Mascaró *et al.* 2022). Indeed, the surfing syngameon hypothesis (Caujapé-Castells 2011) argues that secondary contact and subsequent gene flow in insular habitats among genotypes that may have been previously isolated in the mainland generated hybrid swarms in certain island regions that were critical to the successful colonization of many insular regions. Finally, we cannot discard that centuries of human intervention in the ecosystems of these Macaronesian archipelagos—particularly affecting population number and size in *A. argentea* and *A. gorgonum* (Romeiras *et al.* 2016; Rivers and Fernandes 2017)—could have erased part of their original genetic diversity. However, the comprehensive sampling that includes some of the oldest regions of the islands and many well-conserved habitats ensures that most of the genetic variability within these species has been included in the study.

### Phylogeographic Structure Within the Macaronesian Archipelagos

Recent reviews on anagenetic diversification in oceanic islands have concluded that geographical patterning of genetic variation is unexpected among populations of endemic species originating anagenetically (Stuessy *et al*. 2014; Takayama *et al*. 2015). In contrast, haplotype distribution and spatial genetic analyses obtained in all three species of endemic Macaronesian *Artemisia* showed clear phylogeographic structures (Fig. 2; **[see Supporting Information—Figure S2**]).

According to our time-calibrated phylogenetic inference (Fig. 1), the endemic *Artemisia* species from Madeira archipelago, *A. argentea*, could be the first taxon of the group having started intraspecific diversification. The cladogram we obtained shows that haplotype Hm1 diverged earlier than rest of the haplotypes found in the species. This haplotype Hm1 was exclusively found in eastern islands of the archipelago (i.e. Desertas Islands and Porto Santo), where it is the main haplotype, present in all analysed individuals excepting one (Fig. 2). The western island of Madeira harboured three haplotypes—all of them evolutionary distanced from Hm1—which are private and dominant in each of the three studied population in that island. The geographic structure showed by the spatial genetic analysis **[see Supporting Information—Figure S2A]** also supports that populations of *A. argentea* from Porto Santo and Desertas Islands constitute a differentiated genetic group from Madeira Island populations. A similar phylogeographic structure, separating populations from Madeira and Porto Santo, was also reported in *Euphorbia piscatoria* Aiton (Barres *et al*. 2017). According to our phylogenetic reconstruction (Fig. 1), the early diverging condition of Hm1 could indicate that inter-island colonization of *A. argentea* in this archipelago took place from Porto Santo or Desertas to Madeira in an east-to-west route. The observed higher haplotype diversity in Madeira than in Porto Santo (Table 3) could be explained by the larger physical dimensions and greater topographical complexity of Madeira Island (Stuessy *et al*. 2014). Alternatively, as proposed by Caujapé-Castells *et al*. (2017) for the Canary Islands, the younger island of Madeira could have been colonized later than Porto Santo or Desertas, but still contains high genetic variation because drift and/or selection have not acted yet.

Canary Islands are considered the Macaronesian archipelago hosting the highest levels of taxonomic and genetic diversity (Caujapé-Castells *et al*. 2022), this pattern being generally explained by their proximity to the continent, high geological and topographical complexity as well as the variety of habitats and ecosystems they harbour (Pérez de Paz and Caujapé-Castells 2013). In contrast, according to our results, genetic diversity levels in *A. thuscula* are similar or even slightly lower than in the endemic species from Madeira or Cape Verde archipelagos (Table 3). Specifically, Tenerife has been usually reported as the island of the archipelago hosting the highest levels of genetic diversity, probably due to its large size, central position in the archipelago and vast topographic and geologic complexity (Reyes-Betancort *et al*. 2008; Mairal *et al*. 2015). However, despite being the island with more populations sampled in our study, only one haplotype (Hc2) was recovered among all *A. thuscula* individuals from Tenerife. In contrast, up to three haplotypes were recovered in each of the western islands of La Palma and El Hierro. This phylogeographic result appears contrary to the pattern described in several recent studies dealing with other plant taxa, where central largest islands (mainly Tenerife) are usually inferred as the centres of diversification and as the origin of stepwise dispersal to the smaller islands of La Palma and El Hierro in the west (e.g. Sanmartín *et al*. 2008; Fernández-Mazuecos and Vargas 2011; Vitales *et al*. 2014; Mairal *et al*. 2015). The higher haplotype diversity observed in western islands—four out of the seven Canarian haplotypes being exclusively found in those two islands—also mismatches the east–west (high to low) genetic diversity gradient proposed by Caujapé-Castells (2011) for other plant taxa as well. The contrasting pattern we found might suggest that western islands were the first being colonized by the endemic *Artemisia* species. Alternatively, as commented above for the similar geographic pattern found in the Madeiran archipelago with *A. argentea*, western islands of Canarian archipelago could have been colonized later—from central or eastern islands following the typical east-to-west model—, but still contain high genetic variation because drift and/or selection have not acted yet.

The phylogeographic pattern reported in *A. thuscula* also shows two clearly defined groups: one for eastern islands of Tenerife and Gran Canaria (hosting two haplotypes) and another one for western islands of La Gomera, La Palma and El Hierro (hosting five haplotypes; **[see Supporting Information—Figure S2B]**). This genetic split is also suggested by genome size results, where Tenerife and Gran Canaria populations show the lowest qDNA values of the archipelago (Table 3). Such a clear oceanic barrier between Tenerife and La Gomera has not been commonly found in previous studies of Canarian endemic species with widespread distribution in the archipelago, the proximity between the two islands being usually translated into shared genetic diversity among their populations (e.g. García-Verdugo *et al*. 2009, 2021; Mairal *et al*. 2015; Coello *et al*. 2021). Our results show that—even in species with wind seed dispersal as *Artemisia—*strong population genetic differentiation can be found among two of the closest islands of the archipelago.

Unlike other Macaronesian archipelagos such as the Canary Islands or Madeira, Cape Verde flora has been considerably less studied from a genetic diversity point of view (Romeiras *et al.* 2019). Only a few Cape Verdean endemic plant species (e.g. *Echium*, Romeiras *et al.* 2011; *Campanula jacobaea* C.Sm. ex Hook., Alarcón *et al*. 2013; *Cynanchum daltonii* (Decne.) Liede & Meve, *Globularia amygdalifolia* Webb and *Umbilicus schmidtii* Bolle, Romeiras *et al*. 2015; *Euphorbia tuckeyana* Steud. ex Webb, Barres *et al*. 2017) have been the focus of molecular analyses devoted to study their genetic structure. Notwithstanding this limited number of studies, a clear phylogeographic pattern has been inferred: all those species mainly distributed in western islands of the archipelago (i.e. *Echium*, *C. jacobaea, G. amygdalifolia, U. schmidtii* and *E. tuckeyana*) showed an evident north-south differentiation pattern at the molecular level. Our results in *A. gorgonum*—a species also distributed in western islands of Cape Verde—support as well the same phylogeographic structure. Three related haplotypes were found in *Artemisia* populations from the northern Santo Antão Island (Fig. 2), which constitute a genetic cluster **[see Supporting Information—Figure S2C]**, while three different evolutionarily-close haplotypes were observed in populations from southern islands of Fogo and Santiago, also constituting a separate genetic cluster. Despite the sampling of genome size assessments is limited to one population per island, differences in qDNA values among northern (Santo Antão) and southern (Fogo and Santiago) islands also suggest this geographic pattern (Table 3). As proposed by Romeiras *et al*. (2015), isolation and genetic drift resulting from the fragmented distributions among the northern and southern islands likely explain this phylogeographic structure.

### Anagenetic speciation of endemic Macaronesian *Artemisia*

The endemic Macaronesian *Artemisia* species are unique examples of anagenetic diversification in three different archipelagos occurring within the same evolutionary group. Besides the particularities found in each lineage and archipelago, our results revealed that these insular *Artemisia* species share some remarkable evolutionary patterns. The time-calibrated phylogenetic reconstruction suggested that divergence among the three lineages occurred in a coincidental short period of time during the Pleistocene. Haplotype and genetic differentiation analyses showed similar diversity values among *A. argentea, A. thuscula* and *A. gorgonum*. Clear phylogeographic patterns—showing comparable genetic structuring among groups of islands—were also found in the three archipelagos. Even from the cytogenetic point of view, the three species presented similarly lower genome size values compared to the mainland closely related species *A. arborescens*. These parallelisms between the diversification processes experienced by the endemic *Artemisia* in Madeira, Canary Islands and Cape Verde archipelagos represent a unique opportunity to understand why some island endemic plants fail to radiate in multiple species.

Reproductive features and ecological preferences are usually regarded as key biological attributes to explain adaptive radiation in oceanic islands (García-Verdugo *et al*. 2014; Takayama *et al*. 2018). The small achenes of *Artemisia* present anemochorous dispersal (Vallès and McArthur 2001; Watson *et al*. 2002; Vallès *et al*. 2011), which ensures its long-range transport on a regular basis. Large capacity of seed dispersal may provide good inter- and intra-island colonization capabilities as well as high genetic diversity levels in island endemics (Pérez de Paz and Caujapé-Castells 2013), but may also prevent the genetic isolation of populations, a factor that has been identified as a major driving force in the process of radiation of species in oceanic islands (Losos and Ricklefs 2009; García-Verdugo *et al*. 2014). However, clear phylogeographic structures—in all cases involving inter-island genetic groups that do not share any haplotype—have been found in the plastid DNA data from the three endemic Macaronesian *Artemisia*. These genetic differentiation patterns could suggest that the species are experiencing ongoing evolutionary divergence processes eventually leading to a taxonomic diversification. Alternatively, given that the pollination of *Artemisia* species occurs also through air (Vallès and McArthur 2001; Watson *et al*. 2002; Vallès *et al*. 2011), long-range pollen transport could be contributing more than seed dispersal to the gene flow between populations from different islands, avoiding genetic isolation and speciation.

Low rate of speciation in some island endemics may also be influenced by the type of habitat occupied by these plants (García-Verdugo *et al*. 2014). *Artemisia thuscula* is one of the characteristic elements of the extensive lowland scrub vegetation of the Canary Islands. In Cape Verde, *A. gorgonum* was formerly one of the main species of the scrub vegetation of the islands, and nowadays it is considered in danger of extinction as a consequence of anthropogenic pressures (Romeiras *et al.* 2016). Landscape formation of these Macaronesian *Artemisia* could enable the genetic flow between populations, preventing strong genetic isolation and taxonomic diversification of the species. According to the surfing syngameon hypothesis, evolutionary change in the plant populations that occur in open island areas should be slowed down by introgression following high levels of gene flow among the colonizing individuals (Caujapé-Castells *et al*. 2017). The importance of habitat in the diversification of oceanic *Artemisia* species was already stated by Hobbs and Baldwin (2013), who inferred that intra-archipelago speciation in endemic Hawaiian *Artemisia* was related to niche migration from lower localities of tropical climate to cool and dry high-elevation habitats. In *A. argentea*, morphological differentiation has been observed between plants growing isolated in Madeira summits above 1000 m of elevation (A. Santos, unpublished results) and the rest of the populations of this species occupying—in some cases dominating—coastal sites close to sea level. This potential taxonomic diversification process occurring within Madeira archipelago would support the role of habitat type and niche conservation/migration as driving forces for speciation in insular *Artemisia*.

Overall, we hypothesize that characteristics of seed dispersal, pollen transport and type of habitat in endemic Macaronesian *Artemisia* species—together with their recent evolutionary history as independent lineages—could be key factors to explain the limited speciation ability within this group of plants. Further genetic studies using highly variable nuclear markers (e.g. high-throughput sequencing approaches) would elucidate the role of gene flow via pollen or seeds in the evolutionary history of these island endemics. Additional experiments regarding their reproductive biology (e.g. the occurrence of self-compatibility or self-incompatibility) would also be helpful to fully understand these textbook examples of anagenetic diversification in oceanic islands.

## Supporting Information

The following additional information is available in the online version of this article –

Tables S1-S5 and Figures S1-S4 are provided in the Supplementary Information PDF file.

**Table S1.** Biological traits in endemic Macaronesian *Artemisia* species and *A. arborescens*. Information obtained from the floras of the territories where these species are distributed (see the main text for the references).

**Table S2.** Sampling details of the populations studied from the genetic and cytogenetic point of view.

**Table S3.** GenBank accession numbers of the sequences generated for the populations studied from the genetic point of view.

**Table S4.** Results of analyses of molecular variance (AMOVA) in the geographical and genetic groups of populations defined in the study.

**Table S5.** Nuclear DNA content of the species studied, where the 2C values of the three Macaronesian species (with a representative of each island where the taxa live) listed below are the first obtained in natural populations. Asterisks (*) indicate data from Garcia *et al.* (2006)

**Figure S1.** Values of ΔF_CT_ used to estimate the most likely K from SAMOVA analyses.

**Figure S2.** Geographic distribution of the populations according to the spatial genetic partitioning defined by SAMOVA for K = 2 in (A) *A. argentea*, (B) *A. thuscula* and (C) *A. gorgonum*.

**Figure S3.** Statistical parsimony allelic networks for nuclear (ITS and ETS) phased sequences. Different colours in the pie charts (ribotypes) correspond to the different species. Sequences constituting each inferred ribotype are specified next to each pie chart.

**Figure S4.** Neighbour-net network inferred from nuclear ribosomal DNA sequences. Tip names correspond to the sample codes shown in Table 1.

plad057_suppl_Supplementary_Material

## Data Availability

The data presented in this study are openly available in GenBank at NCBI. GenBank accession numbers are provided in **Supporting Information—Table S3**.

## Notes

**Evolution and Diversity. Chief Editor:** Jeremy Beaulieu
