# Supplementary material for: Parallel anagenetic patterns in endemic Artemisia species from three Macaronesian archipelagos
Source: AoB Plants. 2023 Aug 14;15(4):plad057. doi: 10.1093/aobpla/plad057 (PMC10465267; doi:10.1093/aobpla/plad057)
Supplement: plad057_suppl_Supplementary_Material [file plad057_suppl_supplementary_material.pdf]

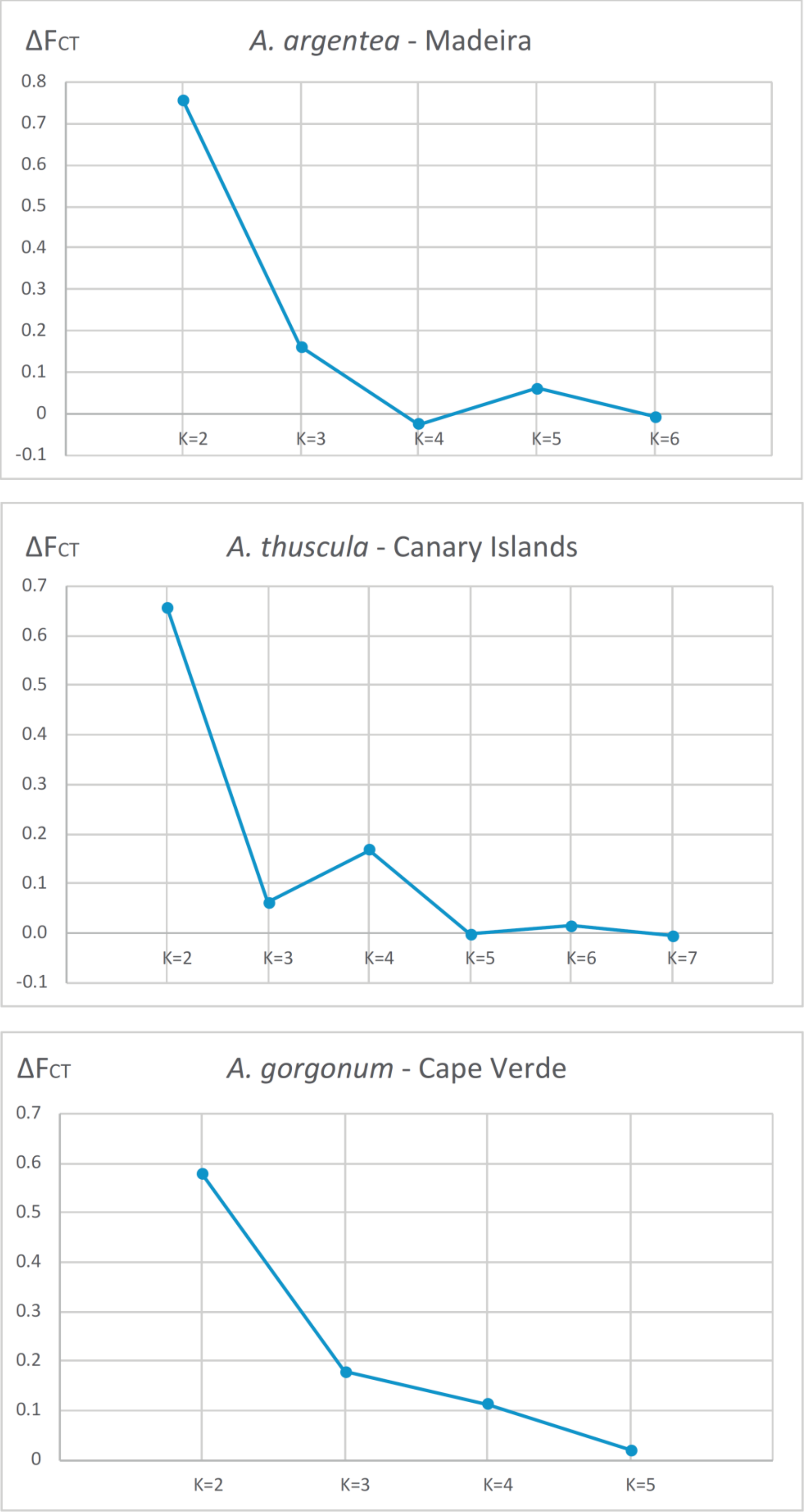

Figure S1. Values of ΔF_{CT} used to estimate the most likely K from SAMOVA analyses.

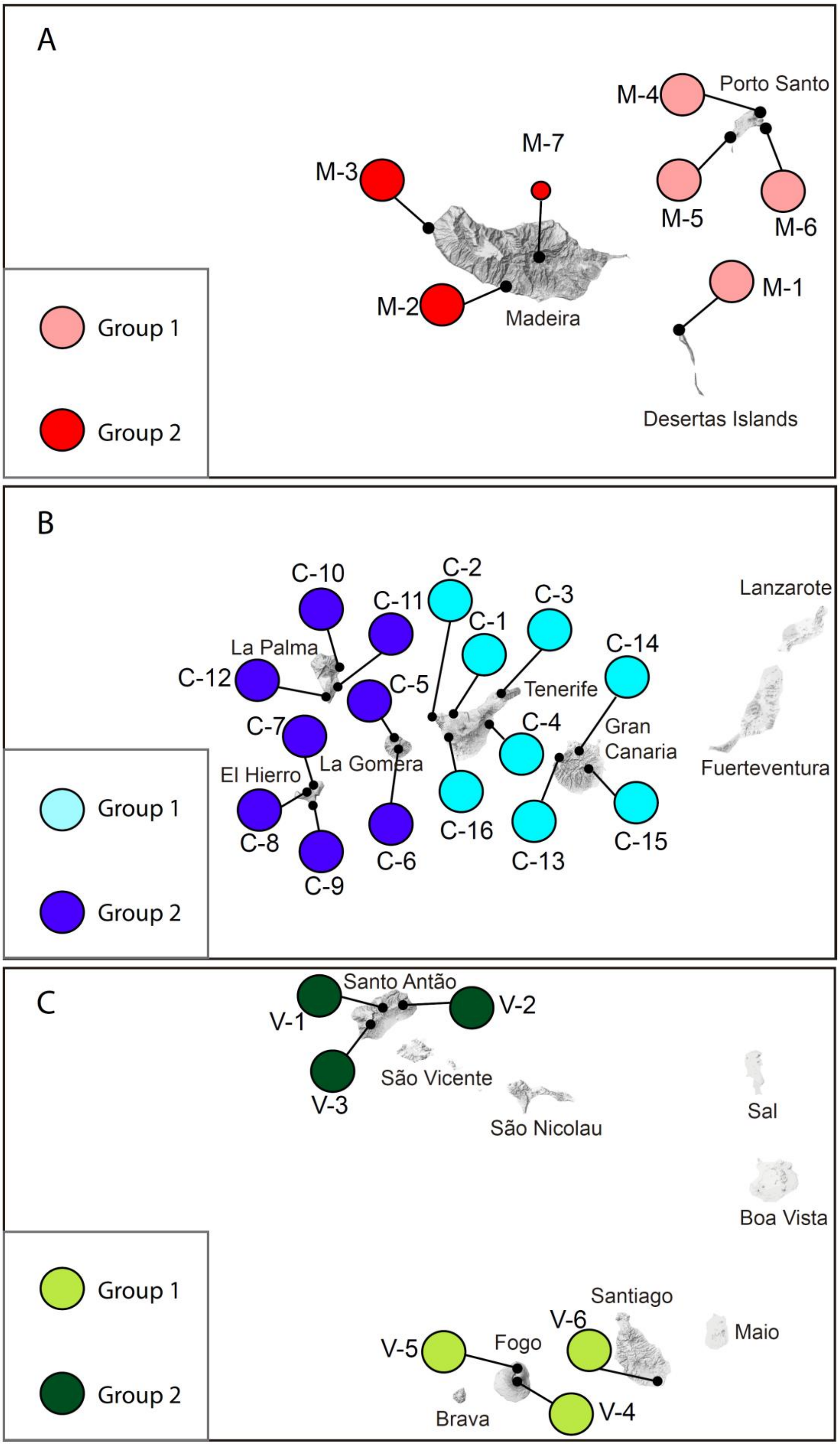

Figure S2. Geographic distribution of the populations according to the spatial genetic partitioning defined by SAMOVA for K = 2 in (A) *A. argentea*, (B) *A. thuscula* and (C) *A. gorgonum*.

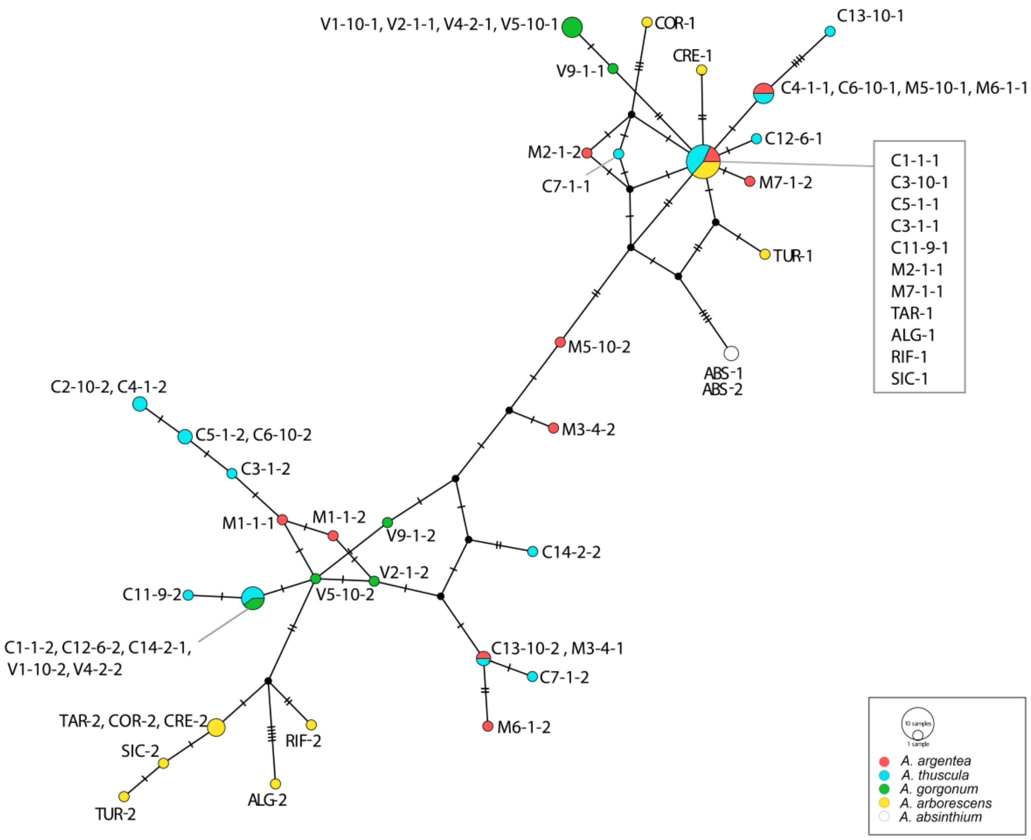

Figure S3. Statistical parsimony allelic networks for nuclear (ITS and ETS) phased sequences. Different colours in the pie charts (ribotypes) correspond to the different species. Sequences constituting each inferred ribotype are specified next to each pie chart.

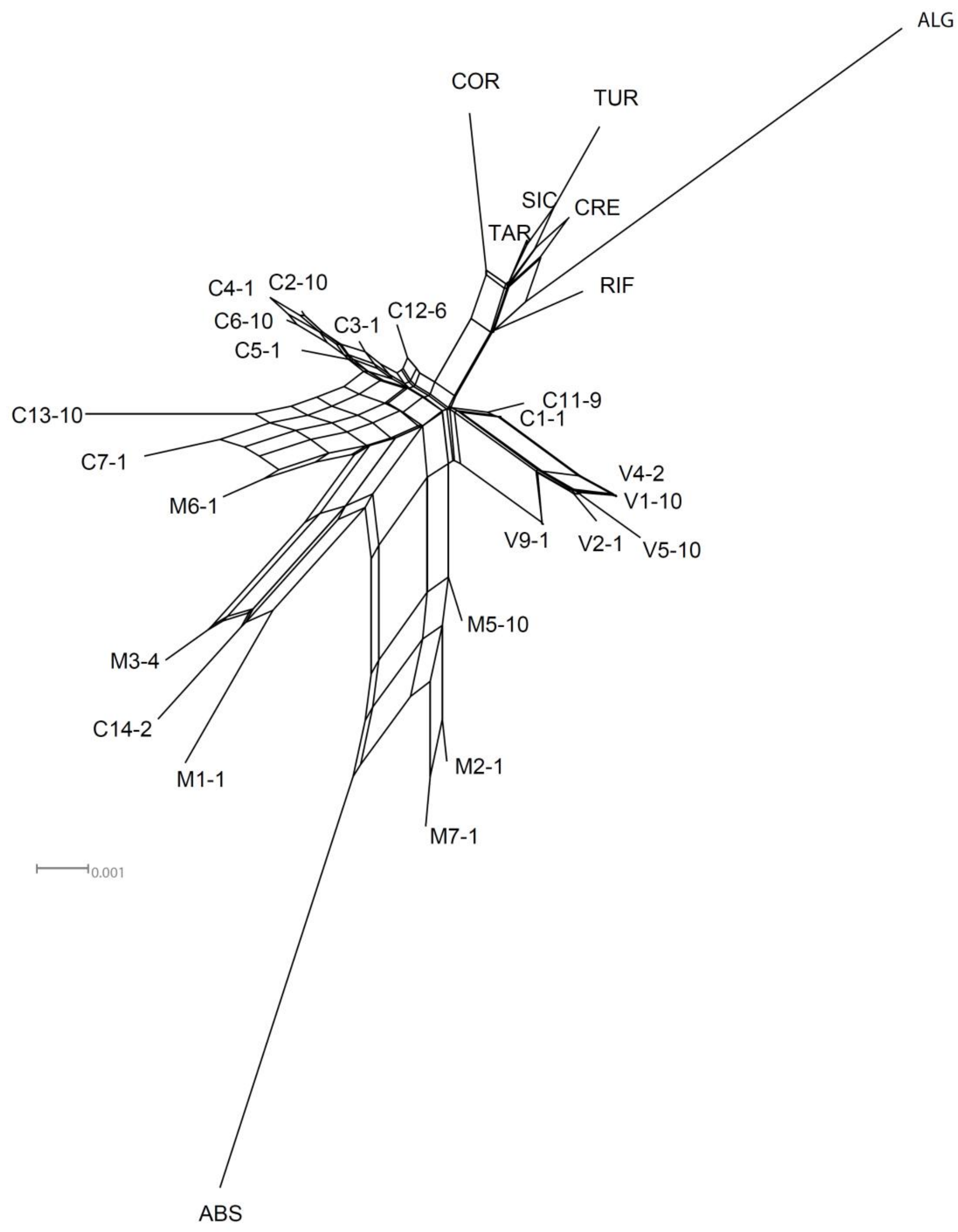

Figure S4. Neighbor-net network inferred from nuclear ribosomal DNA sequences. Tip names correspond to the sample codes showed in Table 1.

Table S1. Biological traits in endemic Macaronesian *Artemisia* species and *A. arborescens*. Information obtained from the floras of the territories where these species are distributed (see the main text for the references).

	A. arborescens	*A. argentea*	*A. thuscula*	*A. gorgonum*
Geographic range	Mediterranean region	Madeira archipelago	Canary Islands	Cape Verde archipelago
Habitat	Maritime scrub	Maritime scrub; mountain peaks[1]	Lowland scrub	Semi-arid to humid scrub
Elevation range	0-600 m	0-300; >1000 m[1]	50-700 m	400-2400 m
Life form	Shrub < 1.5 m	Shrub < 1 m	Shrub < 1 m	Shrub < 2 m
Leaf shape	1-2 pinnatisect	1-2 pinnatisect	2-pinnatisect	2-3 pinnatisect
Leaf size	3-5 cm L	3-6.5(-8) cm L x 3-5.5(-8) cm W	3-7 cm L	8 cm L x 6 cm W
Capitulum color	Brownish	Yellow[2]	Golden to brownish yellow	Yellowish
Capitulum diameter	5-8 mm	3-5 mm[3]	4 mm	-

[1] Isolated populations of *A. argentea* have been found in Madeira Island peaks.

[2] Plants with purple flowers occur among the yellow-flowered populations in Ilhéu de Cima.

[3] In this case, measure referring to the diameter of the involucre

Table S2. Sampling details of the populations studied from the genetic and cytogenetic point of view.

Taxon	Codes	Collection data	Herbarium voucher	Latitude; longitude	N	Haplotype (sample number)
Artemisia thuscula Cav. (*Artemisia canariensis* Less.)	C-1*	Spain, Canary Islands, Tenerife. Icod de los Vinos, road to the San Marcos beach, 70 m. Arnoldo Santos–Guerra & Joan Vallès. 7–XI–2011	BCN 97856	28°22'19.43"N; 16°43'22.72"W	5	Hc2(5)
Artemisia thuscula Cav. (*Artemisia canariensis* Less.)	C-2*	Spain, Canary Islands, Tenerife. Road to the Punta de Teno, 1 km before the location, 15 m. Arnoldo Santos–Guerra & Joan Vallès. 7–XI–2011	BCN 97821	28°20'55.54"N; 16°54'50.81"W	5	Hc2(5)
Artemisia thuscula Cav. (*Artemisia canariensis* Less.)	C-3*	Spain, Canary Islands, Tenerife. Anaga mountains, viewpoint of La Jardina. Arnoldo Santos–Guerra & Joan Vallès. 8–XI–2011	BCN 97823	28°31'57.17"N; 16°17'40.81"W	5	Hc2(5)
Artemisia thuscula Cav. (*Artemisia canariensis* Less.)	C-4*	Spain, Canary Islands, Tenerife. Güimar, viewpoint of Martín. Arnoldo Santos–Guerra & Joan Vallès. 8–XI–2011	BCN 97826	28°17'21.56"N; 16°24'6.39"W	5	Hc2(5)
Artemisia thuscula Cav. (*Artemisia canariensis* Less.)	C-5*	Spain, Canary Islands, La Gomera. Tunel of La Culata, begining of the descent to Vallehermoso, 320 m. Arnoldo Santos–Guerra & Joan Vallès. 9–XI–2011	BCN 97830	28°11'1.58"N; 17°15'16.81"W	5	Hc3(5)
Artemisia thuscula Cav. (*Artemisia canariensis* Less.)	C-6*	Spain, Canary Islands, La Gomera. Benchijigua. Arnoldo Santos–Guerra & Joan Vallès. 10–XI–2011	BCN 97831	28° '35.99"N; 17°13'4.01"W	5	Hc3(5)
Artemisia thuscula Cav. (*Artemisia canariensis* Less.)	C-7*	Spain, Canary Islands, El Hierro. Near the viewpoint of La Peña. Arnoldo Santos–Guerra & Joan Vallès. 11–XI–2011	BCN 97851	27°48'27.09"N; 17°58'46.83"W	5	Hc3(2), Hc4(2), Hc6(1)
Artemisia thuscula Cav. (*Artemisia canariensis* Less.)	C-8	Spain, Canary Islands, El Hierro. Los Llanillos, 3 km west of the village, 310 m. Arnoldo Santos–Guerra & Joan Vallès. 10–XI–2011	BCN 97850	27°45'9.50"N; 18° 2'18.16"W	5	Hc3(4), Hc6(1)
Artemisia thuscula Cav.	C-9	Spain, Canary Islands, El Hierro. In between El Pinar and La Restinga,	BCN 97849	27°38'44.51"N;	5	Hc3(5)

(*Artemisia canariensis* Less.)		430 m. Arnoldo Santos–Guerra & Joan Vallès. 10–XI–2011		17°59'5.57"W		
Artemisia thuscula Cav. (*Artemisia canariensis* Less.)	C-10	Spain, Canary Islands, La Palma. Punta Llana, Martín Luis, 220 m. Arnoldo Santos–Guerra & Joan Vallès. 12–XI–2011	BCN 97848	28°44'23.21"N; 17°44'43.67"W	5	Hc3(5)
Artemisia thuscula Cav. (*Artemisia canariensis* Less.)	C-11*	Spain, Canary Islands, La Palma. Mazo, Las Salemeras, 120 m. Arnoldo Santos–Guerra & Joan Vallès. 13–XI–2011	BCN 97847	28°35'6.02"N; 17°45'40.22"W	5	Hc5(5)
Artemisia thuscula Cav. (*Artemisia canariensis* Less.)	C-12*	Spain, Canary Islands, La Palma. Fuencaliente, neighbourhood of Las Indias, 320 m. Arnoldo Santos–Guerra & Joan Vallès. 14–XI–2011	BCN 97844	28°30'23.52"N; 17°52'4.15"W	5	Hc3(4), Hc7(1)
Artemisia thuscula Cav. (*Artemisia canariensis* Less.)	C-13*	Spain, Canary Islands, Gran Canaria. Andén Verde (in between Agaete and La Aldea), 520 m. Arnoldo Santos–Guerra & Joan Vallès. 14–XI–2011	BCN 97842	28° 1'34.28"N; 15°46'24.95"W	5	Hc2(5)
Artemisia thuscula Cav. (*Artemisia canariensis* Less.)	C-14*	Spain, Canary Islands, Gran Canaria. Agüimes / Ingenio, ravine of Guayadeque, 100 m. Arnoldo Santos–Guerra & Joan Vallès. 15–XI–2011	BCN 97837	27°56'12.37"N; 15°30'34.59"W	5	Hc1(5)
Artemisia thuscula Cav. (*Artemisia canariensis* Less.)	C-15	Spain, Canary Islands, Gran Canaria. Los Tiles de Moya, ravine of Laurel, 480 m. Arnoldo Santos–Guerra & Joan Vallès. 15–XI–2011	BCN 97835	28° 4'41.47"N; 15°35'42.19"W	5	Hc1(1), Hc2(4)
Artemisia thuscula Cav. (*Artemisia canariensis* Less.)	C-16	Spain, Canary Islands, Tenerife. Tejina de Isora, Tejina mountain, 560 m. Arnoldo Santos–Guerra & Joan Vallès. 16–XI–2011	BCN 97854	28°11'12.15"N; 16°45'56.76"W	5	Hc2(5)
Artemisia thuscula Cav. (*Artemisia canariensis* Less.)	C-17$^+$	Spain, Canary Islands, Tenerife. Puerto de la Cruz, path to the beach of Bollullo. Arnoldo Santos-Guerra & Joan Vallès. 8–XI–2011	BCN 97828	28° 24' 51.53"N; 16° 31' 32.45"W	-	-
Artemisia thuscula Cav. (*Artemisia canariensis* Less.)	C-18$^+$	Spain, Canary Islands, La Palma. Fuencaliente, lavas of San Antonio volcano, 450 m. Arnoldo Santos-Guerra & Joan Vallès. 14–XI–2011	BCN 97845	28° 29' 0.72"N; 17° 51' 1.22"W	-	-
Artemisia argentea L'Hér.	M-1*	Portugal, Madeira. Desertas Islands, plain top of Ilhéu Chão, 100 m. Carlos Nóbrega, Arnoldo Santos–Guerra & Joan Vallès. 29–VIII–2012	BCN 99565	32°28'58.00"N; 16°29'30.64"W	5	Hm1(5)

Artemisia argentea L'Hér.	M-2*	Portugal, Madeira. Madeira, near cableway station at Quinta Grande, abandoned vineyards. Francisco Fernandes, Arnoldo Santos–Guerra & Joan Vallès. 1–IX–2012	BCN 99569	32°40'12.36"N; 17° 2'9.17"W	5	Hm4(5)
Artemisia argentea L'Hér.	M-3*	Portugal, Madeira. Madeira, Ponta do Pargo, marine rocks in between the lighthouse and la Casa de Chá. Francisco Fernandes, Arnoldo Santos–Guerra & Joan Vallès. 3–IX–2012	BCN 99574	32°48'45.39"N; 17°15'38.63"W	5	Hm2(5)
Artemisia argentea L'Hér.	M-4	Portugal, Madeira. Porto Santo, early way to Pico Branco, rocky scrubland, 220 m. Francisco Fernandes & Joan Vallès. 5–IX–2012	BCN 99580	33° 5'39.56"N; 16°17'55.20"W	5	Hm1(5)
Artemisia argentea L'Hér.	M-5*	Portugal, Madeira. Porto Santo, Os Morenos, scrub dominated by *Artemisia*, with *Frankenia* and *Atriplex*, 70 m. Francisco Fernandes & Joan Vallès. 5–IX–2012	BCN 99581	33° 2'0.87"N; 16°23'7.70"W	5	Hm1(5)
Artemisia argentea L'Hér.	M-6*	Portugal, Madeira. Ilhéu do Farol (= Ilhéu de Cima = Ilhéu dos Dragoeiros), scrub dominated by *Artemisia* with *Dracaena draco* reintroduced, 100 m. Francisco Fernandes & Joan Vallès. 6–IX–2012	BCN 99583	33° 3'18.70"N; 16°16'57.43"W	5	Hm1(4), Hm3(1)
Artemisia argentea L'Hér.	M-7*	Portugal, Madeira, Madeira, north slope, Pico Cidrão, down to Pico do Gato. Arnoldo Santos & Francisco Fernandes. 19–XI –2014	14648 MADJ	32°44'29.23"N; 16°56'22.98"W	1	Hm5(1)
Artemisia gorgonum Webb	V-1*	Cape Verde, Santo Antão. Natural park of Moroços, Espadaná, 1450 m. Ana Carla Gonçalves, Emitério Ramos, Maria Romeiras, Paulo Silveira & Joan Vallès. 27–I–2013	BCN 101807	17° 6'28.26"N; 25° 4'7.28"W	5	Hv1(5)
Artemisia gorgonum Webb	V-2*	Cape Verde, Santo Antão. Natural park do Topo de Corroa, in between Bolona and Campo Redondo, 1580 m. Ana Carla Gonçalves, Emitério Ramos, Maria Romeiras, Paulo Silveira & Joan Vallès. 28–I–2013	BCN 101806	16°59'35.66"N; 25°14'17.05"W	5	Hv2(4), Hv3(1)
Artemisia gorgonum Webb	V-3	Cape Verde, Santo Antão. Pico da Cruz, slopes, terraces and rocks on the sidelines of the way to the top, near the creek of Covoada Funda, 1350 m. Ana Carla Gonçalves, Emitério Ramos, Maria Romeiras, Paulo	BCN 101805	17° 6'15.65"N; 25° 2'35.59"W	5	Hv3(5)

		Silveira & Joan Vallès. 28–I–2013				
Artemisia gorgonum Webb	V-4*	Cape Verde, Santiago. Praia, Sucupira marketplace, material acquired in a local market, from the Pico da Antónia. Ana Carla Gonçalves, Paulo Silveira& Joan Vallès. 31–I–2013	BCN 101803	14°55'50.18"N; 23°30'45.00"W	5	Hv4(5)
Artemisia gorgonum Webb	V-5*	Cape Verde, Fogo. Natural park do Fogo, Floresta do Monte Velha, road to Montinho, reforested and humid place, 1800 m. Herculano Dinis, Cláudia Fernandes, Maria Romeiras, Paulo Silveira & Joan Vallès. 1–II–2013	BCN 101799	15° 0'14.31"N; 24°21'19.98"W	5	Hv4(3), Hv5(1), Hv6(1)
Artemisia gorgonum Webb	V-6	Cape Verde, Fogo. Natural park do Fogo, little below from the Topo da Bordera, on lapilli, near the “Mi mi sô”, 2050 m. Herculano Dinis, Cláudia Fernandes, Maria Romeiras & Joan Vallès. 3–II–2013	BCN 101801	14°55'42.28"N; 24°21'22.12"W	5	Hv4(5)
Artemisia gorgonum Webb	V-7[+]	Cape Verde, Santiago. São Jorge dos Orgãos, going up to the Pico da Antónia, Longueiras, 550 m. Cultivated plants from Pico da Antónia. Ana Carla Gonçalves, Maria Romeiras, Paulo Silveira, Jorge Tavares & Joan Vallès. 31–I–2013	BCN 101802	15°03'00.0"N; 23°36'17.6"W	-	-
Artemisia gorgonum Webb	V-8[+]	Cape Verde, Fogo. Parque natural do Fogo, Chã das Caldeiras, path towards Monte Velha, at the bottom of a river of solidified lava, 1700 m. Herculano Dinis, Cláudia Fernandes, Maria Romeiras, Paulo Silveira & Joan Vallès. 1–II–2013	BCN 101800	14°58'05.9"N; 24°22'24.3"W	-	-
Artemisia gorgonum Webb	V-9*	Cape Verde, Fogo. Ribeira do Monte Preto, bed of a stream, now dry, but with water in the rainy season. Herculano Dinis, Cláudia Fernandes, Maria Romeiras, Paulo Silveira & Joan Vallès. 2–II–2013	BCN 101798	NA	-	-
Artemisia arborescens L.	TAR*	Spain, Catalonia, Tarragona. Road from Roquetes to Alfara de Carles (road TV–3422), km 8 (near the canal), fence of a house. Teresa Garnatje & Roser Vilatersana. 26–V–2005	BCN 30514	40°51'41.57"N; 0°25'11.81"E	1	H19(5)

Artemisia arborescens L.	ALG*	Algeria, Béjaïa, national park of Gouraya. Joan Vallès. 11–X–2004	BCN 28635	36°46'52.20"N; 4°58'44.48"E	1	H19(1)
Artemisia arborescens L.	COR*	France, Corsica, Bonifaziu, Sotta. Over the walls of the ancient Citadelle. Maria Bosch & Maria R. Orellana. 9–V–2005	BCN 30516	41°33'53.34"N; 9°11'19.64"E	1	H19(1)
Artemisia arborescens L.	RIF*	Morocco, Rif. Cultivated in a private garden. Oriane Hidalgo & Angel Romo. 20–VI–2005	BCN 30560	34°59'35.92"N; 3°59'37.46"W	1	H19(1)
Artemisia arborescens L.	SIC*	Italy, Sicily, Custonaci. On the way out of the town going towards Corino. Joan Vallès. 12–IX–2013	BC 931107	NA	1	H19(1)
Artemisia arborescens L.	CRE*	Greece, Crete. Kalyves beach. Teresa Garnatje & Jordi Luque. 15–VIII–2002	BCN 27513	35°27'06.3"N; 24°10'35.2"E	1	H20(1)
Artemisia arborescens L.	TUR*	Turkey, Hatay, Çevlik, Kral Mezarlarý Mekvii. Cliffs, 10 meters above sea level. Fadime Gumusboga. 7–VI–2005	BCN 30550	36°15'56.82"N; 36° 0'14.82"E	1	H21(1)
Artemisia absinthium L.	ABS*	Spain, Girona, Setcases, near Pont Nou. Teresa Garnatje. 1–VIII–2008	BCN58749	51º20'2.55''N; 13º42'59.15''E	1	H22(1)

* Populations included in the screening analyses of nuclear ribosomal DNA variability; +Populations only studied from the point of view of genome size; N: Number of individuals sequenced for plastid DNA regions

Table S3. GenBank accession numbers of the sequences generated for the populations studied from the genetic point of view.

Taxon	Codes	ndhC-trnV	rpl32-trnL	rps16-trnK	ITS2	ETS
Artemisia thuscula Cav. (*Artemisia canariensis* Less.)	C-1*	OQ556361-OQ556365	OQ556441-OQ556445	OQ556521-OQ556525	OQ557739	OQ578943
Artemisia thuscula Cav. (*Artemisia canariensis* Less.)	C-2*	OQ556366-OQ556370	OQ556446-OQ556450	OQ556526-OQ556530	OQ557740	OQ578944
Artemisia thuscula Cav. (*Artemisia canariensis* Less.)	C-3*	OQ556371-OQ556375	OQ556451-OQ556455	OQ556531-OQ556535	OQ557742	OQ578946
Artemisia thuscula Cav. (*Artemisia canariensis* Less.)	C-4*	OQ556376-OQ556380	OQ556456-OQ556460	OQ556536-OQ556540	OQ557741	OQ578945
Artemisia thuscula Cav. (*Artemisia canariensis* Less.)	C-5*	OQ556381-OQ556385	OQ556461-OQ556465	OQ556541-OQ556545	OQ557743	OQ578947
Artemisia thuscula Cav. (*Artemisia canariensis* Less.)	C-6*	OQ556386-OQ556390	OQ556466-OQ556470	OQ556546-OQ556550	OQ557744	OQ578948
Artemisia thuscula Cav. (*Artemisia canariensis* Less.)	C-7*	OQ556391-OQ556395	OQ556471-OQ556475	OQ556551-OQ556555	OQ557745	OQ578949
Artemisia thuscula Cav. (*Artemisia canariensis* Less.)	C-8	OQ556396-OQ556400	OQ556476-OQ556480	OQ556556-OQ556560	-	-
Artemisia thuscula Cav. (*Artemisia canariensis* Less.)	C-9	OQ556401-OQ556405	OQ556481-OQ556485	OQ556561-OQ556565	-	-
Artemisia thuscula Cav. (*Artemisia canariensis* Less.)	C-10	OQ556406-OQ556410	OQ556486-OQ556490	OQ556566-OQ556570	-	-
Artemisia thuscula Cav. (*Artemisia canariensis* Less.)	C-11*	OQ556411-OQ556415	OQ556491-OQ556495	OQ556571-OQ556575	OQ557746	OQ578950
Artemisia thuscula Cav. (*Artemisia canariensis* Less.)	C-12*	OQ556416-OQ556420	OQ556496-OQ556500	OQ556576-OQ556580	OQ557747	OQ578951
Artemisia thuscula Cav. (*Artemisia canariensis* Less.)	C-13*	OQ556421-OQ556425	OQ556501-OQ556505	OQ556581-OQ556585	OQ557748	OQ578952
Artemisia thuscula Cav. (*Artemisia canariensis* Less.)	C-14*	OQ556426-OQ556430	OQ556506-OQ556510	OQ556586-OQ556590	OQ557749	OQ578953
Artemisia thuscula Cav. (*Artemisia canariensis* Less.)	C-15	OQ556431-OQ556435	OQ556511-OQ556515	OQ556591-OQ556595	-	-
Artemisia thuscula Cav. (*Artemisia canariensis* Less.)	C-16	OQ556436-OQ556440	OQ556516-OQ556520	OQ556596-OQ556600	-	-
Artemisia argentea L'Hér.	M-1*	OQ556601-OQ556605	OQ556632-OQ556636	OQ556663-OQ556667	OQ557754	OQ578954
Artemisia argentea L'Hér.	M-2*	OQ556606-OQ556610	OQ556637-OQ556641	OQ556668-OQ556672	OQ557755	OQ578955
Artemisia argentea L'Hér.	M-3*	OQ556611-OQ556615	OQ556642-OQ556646	OQ556673-OQ556677	OQ557756	OQ578956

Artemisia argentea L'Hér.	M-4	OQ556616-OQ556620	OQ556647-OQ556651	OQ556678-OQ556682	-	-
Artemisia argentea L'Hér.	M-5*	OQ556621-OQ556625	OQ556652-OQ556656	OQ556683-OQ556687	OQ557757	OQ578957
Artemisia argentea L'Hér.	M-6*	OQ556626-OQ556630	OQ556657-OQ556661	OQ556688-OQ556692	OQ557758	OQ578958
Artemisia argentea L'Hér.	M-7*	OQ556631	OQ556662	OQ556693	OQ557759	OQ578959
Artemisia gorgonum Webb.	V-1*	OQ556694-OQ556698	OQ556724-OQ556728	OQ556754-OQ556758	OQ557760	OQ578960
Artemisia gorgonum Webb.	V-2*	OQ556699-OQ556703	OQ556729-OQ556733	OQ556759-OQ556763	OQ557761	OQ578961
Artemisia gorgonum Webb.	V-3	OQ556704-OQ556708	OQ556734-OQ556738	OQ556764-OQ556768	-	-
Artemisia gorgonum Webb.	V-4*	OQ556709-OQ556713	OQ556739-OQ556743	OQ556769-OQ556773	OQ557762	OQ578962
Artemisia gorgonum Webb.	V-5*	OQ556714-OQ556718	OQ556744-OQ556748	OQ556774-OQ556778	OQ557763	OQ578963
Artemisia gorgonum Webb.	V-6	OQ556719-OQ556723	OQ556749-OQ556753	OQ556779-OQ556783	-	-
Artemisia gorgonum Webb	V-9*	-	-	-	OQ557764	OQ578964
Artemisia arborescens L.	TAR*	OQ556340	OQ556347	OQ556354	OQ555318	OQ556333
Artemisia arborescens L.	COR*	OQ556341	OQ556348	OQ556355	OQ555319	OQ556334
Artemisia arborescens L.	ALG*	OQ556342	OQ556349	OQ556356	OQ555320	OQ556335
Artemisia arborescens L.	RIF*	OQ556343	OQ556350	OQ556357	OQ555321	OQ556336
Artemisia arborescens L.	CRE*	OQ556344	OQ556351	OQ556358	OQ555322	OQ556337
Artemisia arborescens L.	TUR*	OQ556345	OQ556352	OQ556359	OQ555323	OQ556338
Artemisia arborescens L.	SIC*	OQ556346	OQ556353	OQ556360	OQ555324	OQ556339
Artemisia absinthium L.	ABS*	OQ581166	OQ581167	OQ581168	OQ557765	OQ581165

* Populations included in the screening analyses of nuclear ribosomal DNA variability

Table S4. Results of analyses of molecular variance (AMOVA) in the geographical and genetic groups of populations defined in the study.

Groups analysed	d.f.	SS	Percentage of variation	*P*
Artemisia argentea - Madeira				
Non-hierarchical AMOVA				
All populations				
Among populations	6	45.116	92.70	< 0.0001
Within populations	24	3.204	7.30	< 0.0001
Total	30	48.319		
Hierarchical AMOVA				
Spatial genetic structure defined by SAMOVA K = 2				
Among groups	1	32.544	75.22	0.03030
Among populations	5	12.572	19.99	0.00098
Within populations	24	3.204	4.79	< 0.0001
Total	30	24		
Geographic groups (islands)				
Among groups	2	32.611	60.48	0.07038
Among populations	4	12.505	33.42	0.00098
Within populations	24	3.204	6.10	< 0.0001
Total	30	48.319		
Artemisia thuscula – Canary Islands				
Non-hierarchical AMOVA				
All populations				
Among populations	15	35.546	84.90	< 0.0001
Within populations	64	5.211	15.10	< 0.0001
Total	79	40.757		
Spatial genetic structure defined by SAMOVA K = 2				
Among groups	1	21.372	65.54	< 0.0001
Among populations	14	14.174	23.98	< 0.0001
Within populations	64	5.211	10.48	< 0.0001
Total	79	40.757		
Spatial genetic structure defined by SAMOVA K = 2				
Among groups	4	25.796	59.49	< 0.0001
Among populations	11	9.750	26.90	< 0.0001
Within populations	64	5.211	13.61	< 0.0001
Total	79	40.757		

Table S4 (cont.). Results of analyses of molecular variance (AMOVA) in the geographical and genetic groups of populations defined in the study.

Groups analysed	d.f.	SS	Percentage of variation	*P*
Artemisia gorgonum – Cape Verde				
Non-hierarchical AMOVA				
All populations				
Among populations	5	14.809	85.02	< 0.0001
Within populations	24	2.419	14.98	< 0.0001
Total	29	17.228		
Hierarchical AMOVA				
Spatial genetic structure defined by SAMOVA K = 2				
Among groups	1	9.004	57.58	0.09189
Among populations	4	5.805	30.89	< 0.0001
Within populations	24	2.419	11.53	< 0.0001
Total	29	17.228		
Geographic groups (islands)				
Among groups	2	9.071	38.19	0.13059
Among populations	3	5.738	48.36	0.0001
Within populations	24	2.419	13.45	< 0.0001
Total	29	17.228		

Table S5. Nuclear DNA content of the species studied, where the 2C values of the three Macaronesian species (with a representative of each island where the taxa live) listed below are the first obtained in natural populations. Asterisks (*) indicate data from Garcia *et al.* (2006)

Taxa	Codes	Location	2C (pg)[a]
A. argentea	M-1	Portugal, Madeira, Desertas Islands, Ilhéu Chão	10.25 (0.23)
A. argentea	M-2	Portugal, Madeira, Madeira	10.24 (0.06)
A. argentea	M-4	Portugal, Madeira, Porto Santo	10.09 (0.21)
A. argentea	M-6	Portugal, Madeira, Porto Santo, Ilhéu de Cima	10.07 (0.36)
A. thuscula	C-17	Spain, Canary Islands, Tenerife	10.02 (0.30)
A. thuscula	C-6	Spain, Canary Islands, La Gomera	10.22 (0.21)
A. thuscula	C-9	Spain, Canary Islands, El Hierro	10.24 (0.30)
A. thuscula	C-18	Spain, Canary Islands, La Palma	10.07 (0.29)
A. thuscula	C-15	Spain, Canary Islands, Gran Canaria	9.91 (0.11)
A. gorgonum	V-3	Cape Verde, Santo Antão	10.27 (0.05)
A. gorgonum	V-7	Cape Verde, Santiago	10.60 (0.18)
A. gorgonum	V-8	Cape Verde, Fogo	10.69 (0.10)
A. arborescens	*	Algeria, Gouraya	11.46 (0.09)
A. arborescens	*	Algeria, Essais Hamma	11.32 (0.08)
A. arborescens	*	France, Corsica	11.37 (0.12)
A. arborescens	*	France, Paris	10.97 (0.15)
A. arborescens	*	France, Porquerolles	11.17 (0.13)
A. arborescens	*	France, El Portús	10.37 (0.17)
A. arborescens	*	Greece, Corfu	10.67 (0.08)
A. arborescens	*	Greece, Crete	11.43 (0.11)
A. arborescens	*	Greece, Rhodes	11.22 (0.11)
A. arborescens	*	Italy, Sardinia	11.30 (0.19)
A. arborescens	*	Italy, Sicily	11.41 (0.07)
A. arborescens	*	Morocco, Rif	11.07 (0.07)
A. arborescens	*	Spain, Alacant, La Encina	11.22 (0.17)

A. arborescens	*	Spain, Balearic Islands, Formentera (1)	11.20 (0.05)
A. arborescens	*	Spain, Balearic Islands, Formentera (2)	11.28 (0.15)
A. arborescens	*	Spain, Balearic Islands, Mallorca	11.61 (0.15)
A. arborescens	*	Spain, Balearic Islands, Mallorca	11.15 (0.06)
A. arborescens	*	Spain, Balearic Islands, Menorca, Maó	11.61 (0.23)
A. arborescens	*	Spain, Catalonia, Barcelona, nursery “Tres Pins”	10.74 (0.24)
A. arborescens	*	Spain, Catalonia, Barcelona, Botanic Garden	10.85 (0.15)
A. arborescens	*	Spain, Botanic Garden of Madrid	10.80 (0.13)
A. arborescens	*	Spain, Catalonia, Besalú	10.35 (0.05)
A. arborescens	*	Spain, Catalonia, Roquetes	11.23 (0.10)
A. arborescens	*	Spain, Valencia, nursery “Pro Agri”	11.15 (0.23)
A. arborescens	*	Turkey, Samandag	11.18 (0.11)

[a] 2C nuclear DNA content (mean value ± standard deviation)
